# Mobile Colistin Resistance Genetic Determinants of Non-Typhoid *Salmonella enterica* Isolates from Russia

**DOI:** 10.3390/microorganisms9122515

**Published:** 2021-12-04

**Authors:** Konstantin V. Kuleshov, Anastasia S. Pavlova, Elizaveta D. Shedko, Yulia V. Mikhaylova, Gabriele Margos, Sabrina Hepner, Igor V. Chebotar, Elena V. Korneenko, Alexander T. Podkolzin, Vasiliy G. Akimkin

**Affiliations:** 1 Central Research Institute of Epidemiology, 111123 Moscow, Russia; nasty-pavlov@yandex.ru (A.S.P.); hardcandy.hd@gmail.com (E.D.S.); yulka_ivashka@mail.ru (Y.V.M.); lennatta@yandex.ru (E.V.K.); podkolzin@pcr.ru (A.T.P.); vgakimkin@yandex.ru (V.G.A.); 2 Federal State Budget Scientific Institution “Federal Scientific Center VIEV”, 109428 Moscow, Russia; 3 German National Reference Centre for Borrelia, Bavarian Health and Food Safety Authority, Veterinärstr. 2, 85764 Oberschleissheim, Germany; gmargos1@gmail.com (G.M.); sabrina.hepner@lgl.bayern.de (S.H.); 4 Laboratory of Molecular Microbiology, Pirogov Russian National Research Medical University, 117997 Moscow, Russia; nizarnn@yandex.ru

**Keywords:** whole genome sequencing, antibiotic resistance, *Salmonella* Enteritidis, *Salmonella* Typhimurium, *Salmonella* Bovismorbificans, colistin resistance, *mcr-1*, *mcr-9*

## Abstract

Polymyxin resistance, determined by *mcr* genes located on plasmid DNA, currently poses a high epidemiological threat. Non-typhoid *Salmonella* (NTS) are one of the key pathogens causing diarrheal diseases. Here, we report the isolation and whole genome sequencing of multidrug colistin-resistant/susceptible isolates of non-typhoid *Salmonella enterica* serovars carrying *mcr* genes. Non-typhoid strains of *Salmonella enterica* subsp. *enterica* were isolated during microbiological monitoring of the environment, food, and diarrheal disease patients between 2018 and 2020 in Russia (*n* = 586). *mcr-1* genes were detected using a previously developed qPCR assay, and whole genome sequencing of *mcr* positive isolates was performed by both short-read (Illumina) and long-read (Oxford Nanopore) approaches. Three colistin-resistant isolates, including two isolates of *S*. Enteritidis and one isolate of *S*. Bovismorbificans, carried the *mcr-1.1* gene located on IncX4 and IncI2 conjugative plasmids, respectively. The phenotypically colistin-susceptible isolate of *S*. Typhimurium carried a *mcr-9* gene on plasmid IncHI2. In conclusion, we present the first three cases of *mcr* gene-carrying NTS isolates detected in Russia with both outbreak and sporadic epidemiological backgrounds.

## 1. Introduction

Non-typhoid strains of *Salmonella enterica* (NTS) contribute significantly to the incidence of intestinal infections worldwide [1]. Globally, there are more than 90 million cases of *Salmonella* gastroenteritis each year, resulting in 155,000 deaths [2]. Despite a trend towards a decrease in salmonellosis, it is still the most frequently recorded foodborne zoonosis in Russia. The annual incidence rate of salmonellosis in Russia decreased from 36.6 per 100,000 population in 2012 to 22.0 per 100,000 in 2017, but gradually increased in 2018 and 2019 up to 24.2 per 100,000. In 2020 and 2021, the annual incidence rate of salmonellosis decreased to 14.7 per 100,000 population [3]. During that time period, the prevailing serotypes in the etiological structure of morbidity remained *Salmonella enterica* subsp. *enterica* serotype Enteritidis and serotype Typhimurium [4].

The epidemiological and clinical relevance of NTS is determined by the genomic plasticity that underlies the adaptation to a wide host range, and the emergence of *Salmonella* strains with novel resistant profiles, in particular, the emergence of strains with plasmid-mediated colistin resistance [5,6,7]. Importantly, in clinical practice, colistin is not used for the treatment of salmonellosis but it is a last resort drug in the treatment of infections associated with antibiotic-resistant Gram-negative bacteria and classified by the World Health Organization as ”critically important.” [8] Thus, *Salmonella* strains with plasmid encoding genes that confer resistance to colistin might be a potentially perilous reservoir for resistance determinants that can be moved via horizontal gene transfer among Gram-negative bacteria.

The first report on a plasmid-located *mcr-1* gene in colistin-resistant *Escherichia coli,* obtained from livestock and patients in China, was made by Lui et al. in 2015 [9]. Subsequently, worldwide dissemination of *mcr-1* positive strains in *Enterobacteriaceae* was shown [10]. To date, NTS strains of different serovars harboring *mcr* variants have been isolated from the environment, animals, and humans in various countries, indicating that such NTS may spread *mcr* genes into diverse environmental niches worldwide [7].

Most *mcr-*carrying plasmids belong to the incompatibility plasmid group Inc [11]. A main feature of plasmids belonging to the Inc group is the presence of several toxin–antitoxin systems which enable a high level of plasmid gene expression, and have resistance genes often located under the same promoter [12,13]. Among the studied plasmids with *mcr* genes, the most common are IncX4 (35.2%), IncI2 (34.7%), and IncHI2 (20.5%), with the IncI2 group prevailing in Asia (65.8%) and IncHI2 in Europe (73.3%) [14]. It is important to highlight that Inc plasmids were shown to carry both *mcr* and β-lactamase genes simultaneously [15,16]. In 77.8% of the studied IncHI2 plasmids and 37.9% of the IncI2 ones, the insertion sequence IS*Apl1* was detected near the *mcr* gene, yet it was always absent in IncX4 plasmids [14]. The most common elements that are found in the *mcr* cassette are the PIN-forming sequence *hp*, *pilli* folding protein IV type *pilP*, and the secretion system proteins IV type *virD4* and *virB4* [6,17].

Data from Russia of NTS strains circulating in the environment that carry genetic determinants of colistin resistance have so far been lacking. The aim of our study was to characterize the first four cases of *mcr* gene-carrying NTS isolates detected in Russia with both outbreak and sporadic epidemiological background.

## 2. Materials and Methods

### 2.1. Bacterial Isolation and Identification

The collection included *Salmonella* strains collected by the All-Russia Reference Center of Salmonellosis during the national Salmonellosis surveillance program. In general, the collection of isolates (*n* = 586) included strains derived from humans (*n* = 293, 58.6%), food (*n* = 208, 35.4%), environmental samples (*n* = 81, 13.8%), and animals (*n* = 3, 0.5%). The *Salmonella* isolated from humans included 139 representative isolates from 59 salmonellosis outbreaks (approximately two isolates from each outbreak) and 154 isolates from sporadic cases. *S.* Enteritidis was the most prevalent serotype among the outbreak strains (74.8%).

All isolates were screened using McConkey medium. Individual colonies were used for re-identification and serotyping. Subsequently, each isolate was subjected to serological characterization according to the Kauffmann–White scheme using polyclonal (Petsal, St. Petersburg, Russia) and monoclonal antibodies (Sifin, Berlin, Germany).

### 2.2. Antibiotic Susceptibility Testing

Antibiotic susceptibility was tested by determining the minimum inhibitory concentration (MIC) for antibiotic susceptibility using GI and GII Mikrolatest^®^SensiLaTest MIC panels (Erba Lachema, Brno, Czech Republic). *Escherichia coli* (ATCC 25922) was used as an internal quality control. Isolates were tested on 19 antibiotics grouped into ten classes. Ssusceptibility interpretation criteria were used in accordance with the European Committee on Antimicrobial Susceptibility (EUCAST) interpretative criteria [18]. The double-disk synergy test (DDST) was employed to confirm the extended-spectrum beta-lactamase (ESBL) production using antimicrobial disks of ceftazidime (30 μg), cefepime (30 μg), and amoxicillin-clavulanic acid (20/10 μg). Expansion of the indicator cephalosporin inhibition zone towards the amoxicillin-clavulanic acid disk was considered indicative of ESBL production [19].

### 2.3. Real-Time PCR Assays for mcr-1

*Salmonella* isolates were tested for the presence of *mcr-1* group genes. DNA extraction and real-time polymerase chain reaction (qPCR) was performed using the PCR-kit «AmpliSens^®^ MDR MCR-1-FL» (Central Research Institute of Epidemiology, Moscow, Russia). The qPCR assay was performed on a Rotor-Gene^®^ 6000 (Corbett Research, Mortlake, Australia). Data obtained with all used reagent kits was analyzed using the RotorGene 6000 v1.8 software, according to the manufacturer’s instructions.

### 2.4. Whole-Genome Sequencing and Assembling

*Salmonella* isolates were subjected to HiSeq (Illumina, San Francisco, CA, USA) and MinION (Oxford Nanopore, Oxford, UK) sequencing technology. Genomic DNA purification was performed using a DNeasy^®^ Blood & Tissue Kit (Qiagen, Hilden, Germany), according to the manufacturer’s instructions. The purified genomic DNA was used for both whole genome sequencing technologies (Illumina and Oxford Nanopore). The libraries for Illumina sequencing were prepared using the Nextera XT DNA Library Prep Kit (Illumina, USA), Index Kit (Illumina, USA), and Reagent Kit (Illumina, USA). The sequencing was performed using a HiSeq 2500 instrument that produced 2 × 250 base pair (bp) paired-end reads.

The DNA library for MinION sequencing was prepared using the Native Barcoding Kit 1D (EXP NBD104, Oxford Nanopore) and Ligation Sequencing Kit 1D (SQK-LSK109, Oxford Nanopore, Oxford, UK), according to the manufacturer’s protocol, then sequenced using an R9 SpotON flow cell (FLO-MIN106). The raw FAST5 reads were basecalled using the Guppy Basecalling Software v3.4.4. The reads were then subsequently trimmed by Porechop (https://github.com/rrwick/Porechop) (accessed on 1 November 2020) with the flag ‘discard_middle,’ and filtered on the quality and read length by Nanofilt. The quality threshold was defined as the median quality of initial sets of raw long reads of a particular isolate, while the minimum read length was set to 1000 bp. Accession numbers for the short-read and long-read data for each isolate are available under the following accession numbers: SLR1_8250 − SRR12342988 (long-reads), SRR12342993 (short-reads), SLR1_8245 − SRR15212457, SRR15212459, SLR1_7627 − SRR12342987, SRR12342992, SLR1_8094 − SRR12342986, SRR12342991.

### 2.5. Bioinformatic Analysis

Unicycler v0.4.2 [20] was used to generate a hybrid assembly, combining the long reads (Oxford Nanopore) and short reads (Illumina). Independently, Canu v1.8 [21] assemblies using only the Oxford Nanopore long reads, and subsequent dot-plot analyses using Flexidot [22], were performed to compare assembly results (Unicycler versus Canu). The comparison parameter was the topology and approximate length of the assembled chromosomes and plasmids.

The sequenced genomes were submitted to GenBank and annotated by the prokaryotic annotation pipeline (PGAP). These genomes were automatically imported to the NCBI Pathogen Detection system (https://www.ncbi.nlm.nih.gov/pathogens/) (accessed on 29 November 2021) and to EnteroBase (https://enterobase.warwick.ac.uk/species/index/senterica) (accessed on 29 November 2021). Using EnteroBase’s information, we could identify the sequence types (ST) defined by Achtman’s seven gene MLST scheme, as well as the eBURST groups (eBG) for each strain [23]. These systems also provided results by searching for clonally-related isolates via incorporated bioinformatic pipelines. The NCBI Pathogen Detection system used an SNP-based approach, while EnteroBase used a core genome MLST (cgMLST). It should be noted that in the SNP-based and core genome MLST comparative analysis we also included an isolate of *S.* Enteritidis SLR1_8239 (accession numbers for the short-read SRR15212458), which originated from the same outbreak as SLR1_8245, to demonstrate clonal relationships between isolates.

Each assembled genome was submitted for analyses using the MOB-suite v3.0.0 [24] and PlasmidFinder v1.3 [25] to define replicon types, and to predict mobility for the assembled plasmids.

Screening for antimicrobial resistance (AMR) genes and point mutations were performed by several approaches using ResFinder v4.0 [26] and AMRFinderPlus v3.6.10 [27].

To determine whether differences may exist between the plasmids harboring *mcr* genes, annotated sequences of plasmids were visualized and compared in the Easyfig software v.2.1 [28] or BLAST Ring Image Generator (BRIG) [29].

## 3. Results

### 3.1. Isolates Description and Antimicrobial Susceptibility Testing

During microbiological monitoring of 85 federal subjects of the Russian Federation between 2018 and 2020 by the All-Russian Salmonella Reference Laboratory, NTS isolates (*n* = 586) were collected from the environment, food, and patients. All isolates were subjected to antibiotic susceptibility testing, as well as screened for the presence of *mcr*-1-type by a specific qPCR. In this screen, only three isolates (*S.* Enteritidis (*n* = 2), *S.* Bovismorbificans (*n* = 1)) were shown to carry an *mcr-1* gene (Table 1). In our study, we also included an isolate, SLR1_8094, representing a *S*. Typhimurium with an *mcr-9* gene, which was isolated from a sporadic case in 2019. The presence of an *mcr*-9 gene in its genome was discovered during a whole genome sequencing study of *Salmonella* isolates circulating in Russia, which were characterized as a multidrug-resistant phenotype.

While the *mcr-1* positive *S*. Enteritidis SLR1_8250 and *S*. Bovismorbificans SLR1_7627 strains were isolated from sporadic cases, it should be noted that the *mcr-1* positive strain SLR1_8245 (*S*. Enteritidis) was a representative isolate from an outbreak of salmonellosis with 239 affected people in El’ban village in the Khabarovsk Territory.

Isolates carrying the *mcr-1* gene are characterized by high MIC values to colistin (MIC 8 mg/L) in antimicrobial susceptibility testing (AST). On the contrary, the isolate with the *mcr-9* gene showed no resistance to colistin (MIC 1 mg/L) (Table 2). SLR1_8094 produced ESBL which was confirmed by DDST. Moreover, according to our data, only isolate SLR1_8250 was resistant to a single drug—namely colistin—while the other three isolates showed a multidrug-resistant (MDR) phenotype defined as resistance to drugs of at least three different antimicrobial classes. An extended spectrum of resistance to 11 antimicrobial agents, representing seven antimicrobial classes, was detected in the monophasic variant *S*. Typhimurium SLR1_8094 (Table 2).

### 3.2. Multilocus Sequence Typing and WGS-Typing

The genomes of the sequenced isolates related to the ST and eBURST groups that correlated with the serotyping results, according to the analysis in EnteroBase using Achtman’s seven gene MLST scheme [23]. The *S*. Enteritidis isolates SLR1_8250 and SLR1_8245 belonged to ST-11 and eBG-4; the *S.* Bovismorbificans isolate SLR1_7627 belonged to ST-142 and eBG-34; and the monophasic variant of *S.* Typhimurium, isolate SLR1_8094, was related to ST-34 and eBG-1.

Although the seven gene MLST scheme [23] enables the determination of serovars of *Salmonella* isolates, it is not suitable for the determination of the phylogenetic positions of, and relatedness between, isolates. For such analyses, core genome analyses (SNP analyses) and core genome MLST are better suited. Thus, to further investigate the phylogenetic positions of our isolates in a global context, we used data publicity available in the NCBI Pathogen Detection system (https://www.ncbi.nlm.nih.gov/pathogens/), which contains 398,518 genomes of *Salmonella* (accessed on 10 October 2021), as well as data in EnteroBase (https://enterobase.warwick.ac.uk/species/index/senterica), which contains 326,421 genomes of *Salmonella* (accessed on 10 October 2021). It should be noted that historical Russian isolates of *S.* Enteritids collected from outbreaks and sporadic cases during 2012–2020 were previously submitted to the NCBI Pathogen Detection system under bioproject PRJNA484865 (including SRL1_8239). Although the new Russian isolates investigated in the present study matched the ST of the seven gene MLST scheme (see above), the more detailed core genome analyses (SNP analyses and core genome MLST) did not reveal any close relationships to each other or to other isolates, except for the match between isolates SLR1_8245 and SLR1_8239 (which originated from the same outbreak); all other studied sporadic and outbreak isolates formed unique clades (orphans) differing by at least 50 SNP to other isolates in the NCBI Pathogen Detection system, or showed at least 10 allele differences by core genome MLST in EnteroBase. (Supplementary Figures S1–S4).

### 3.3. Related Genetic Determinants of Salmonella Isolates to Antimicrobial Susceptibility

These four isolates were subjected to whole genome sequencing (WGS) using Illumina and Oxford Nanopore sequencing technology. Combining different WGS platforms provides the opportunity to reconstruct accurately all genomic elements, and to define the locations of plasmid-encoded genes responsible for colistin resistance, as well as additional sets of resistance genes (Table 3). Thus, we applied the PlasmidFinder and MOB-suite pipelines and the results are detailed below (Table 3).

#### 3.3.1. Salmonella Enteritidis SLR1_8250 and SLR1_8245

**Figure 1:**
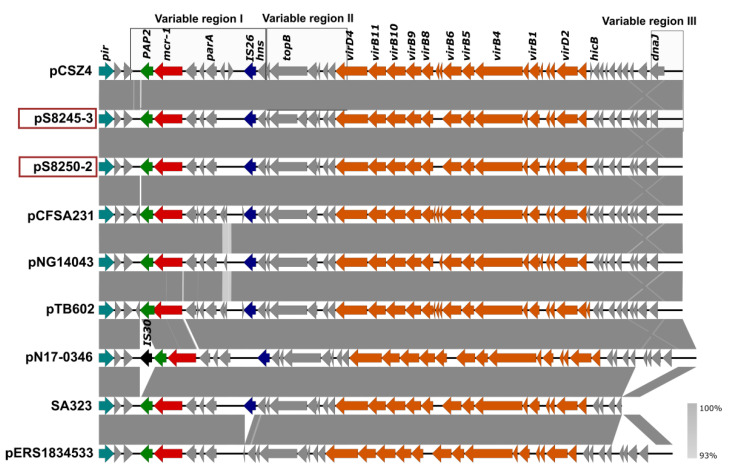
Comparison of the IncX4 plasmids from SLR1_8245, SLR1_8250, and *Salmonella* isolates with previously sequenced IncX4. GenBank accession numbers for these plasmids are listed in Supplementary Table S1. Arrows represent the position of open reading frames. Regions of >99% identity are indicated in dark grey between adjacent plasmid sequences. Genes associated with the *tra* and *pil* loci are colored orange, replication associated genes are colored turquoise, antibiotic resistance genes are colored red, the *PAP2* gene is colored green, insertion sequences are colored blue and black, and other genes are colored gray. The previously defined variable regions I, II, and III [30] were marked by square boxes.

*S.* Enteritidis SLR1_8250 carried three plasmid types (Table 3), namely, a non-mobilizable 60 kb plasmid pS8250-1 of the IncFII(S)/IncFIB(S) type, a mobilizable small 2 kb plasmid pS8250-3 of the ColpVC type, and the conjugative plasmid pS8250-2 of IncX4 type (33 kb). The last one contains the phosphoethanolamine transferase gene (*mcr-1.1,* red arrow in Figure 1), which was located upstream (4-5 kb) of a mobile genomic element of the IS*26*-family. However, there was no copy of the IS*Apl1* insertion sequence flanking the *mcr-1.1* gene. The sequence of a *PAP2* family protein that is associated with *mcr-1.1* was located directly downstream of *mcr-1.1* (green arrow in Figure 1). This IncX4 plasmid also has a standard plasmid backbone containing sequences that are involved in plasmid replication, maintenance, and transfer, as well as genes responsible for the toxin–antitoxin system.

The *S.* Enteritidis SLR1_8245 outbreak strain had three plasmids pS8245-1 (IncFII(S)/IncFIB(S); 60kb), pS8245-2 (IncX1; 57kb), and pS8245-3 (IncX4; 33kb) (Table 3). The *catA1* and *tet(A)* genes responsible for resistance to amphenicols and tetracyclines were located on the IncX1 plasmid. Plasmid pS8245-3 (IncX4) carried the *mcr-1.1* and was similar with 100% coverage and 100% sequence identity to the IncX4 plasmid of isolate SLR1_8250 (Figure 1).

Compared to the non-redundant collection of sequences (NT) representing genomes in the NCBI GenBank database the complete IncX4 sequence was 99% identical to and had a coverage from 92–100% of the IncX4 plasmids of *E. coli* (78 BLAST hits), *K. pneumonia* (12 BLAST hits), and *S. enterica* (11 BLAST hits), showing the prevalence of such a type of IncX4 plasmid among different species of *Enterobacteriaceae*. Comparative analysis with a set of previously sequenced IncX4 plasmids showed a high similarity level (100% coverage and 99.98% sequence identity) of pS8245-3 and pS8250-2 IncX4 plasmids to a so-called epidemic IncX4 plasmid pCSZ4 [30]. The similar IncX4 plasmids pCFSA231, pNG14043, and pTB602 have also been found in other NTS isolates [31,32] (Figure 1, Supplementary Table S1).

#### 3.3.2. Salmonella Bovismorbificans SLR1_7627

The MDR colistin-resistant strain *S*. Bovismorbificans SLR1_7627 isolated from human samples in 2018 carried four plasmids of which only two carried resistance genes (Table 3). The IncI2 conjugative 64 kb plasmid (pS7627-2) included *mcr-1.1,* while additional resistance genes were located on the 233 kb non-mobilizable plasmid pS7627-1 of the IncHI2/IncQ1 type.

The results of the AST correlated well with identified genes responsible for resistance to antibiotics (Table 2 and Table 3). Trimethoprim-sulfamethoxazole resistance is explained by the presence of *sul1*, *sul2*, and *dfrA1* genes. The resistance of the isolate to tetracycline is confirmed by presence of a *tet(A)* gene. As well, the identified *blaTEM-1B* gene enables resistance to penicillins. However, the roles of genes *aadA1, aph(6)-Id,* and gene *aph(3’’)-Ib,* which encode proteins for streptomycin and spectinomycin resistance and resistance to kanamycin, respectively, could not be confirmed due to the absence of these antimicrobial agents in the panel.

**Figure 2:**
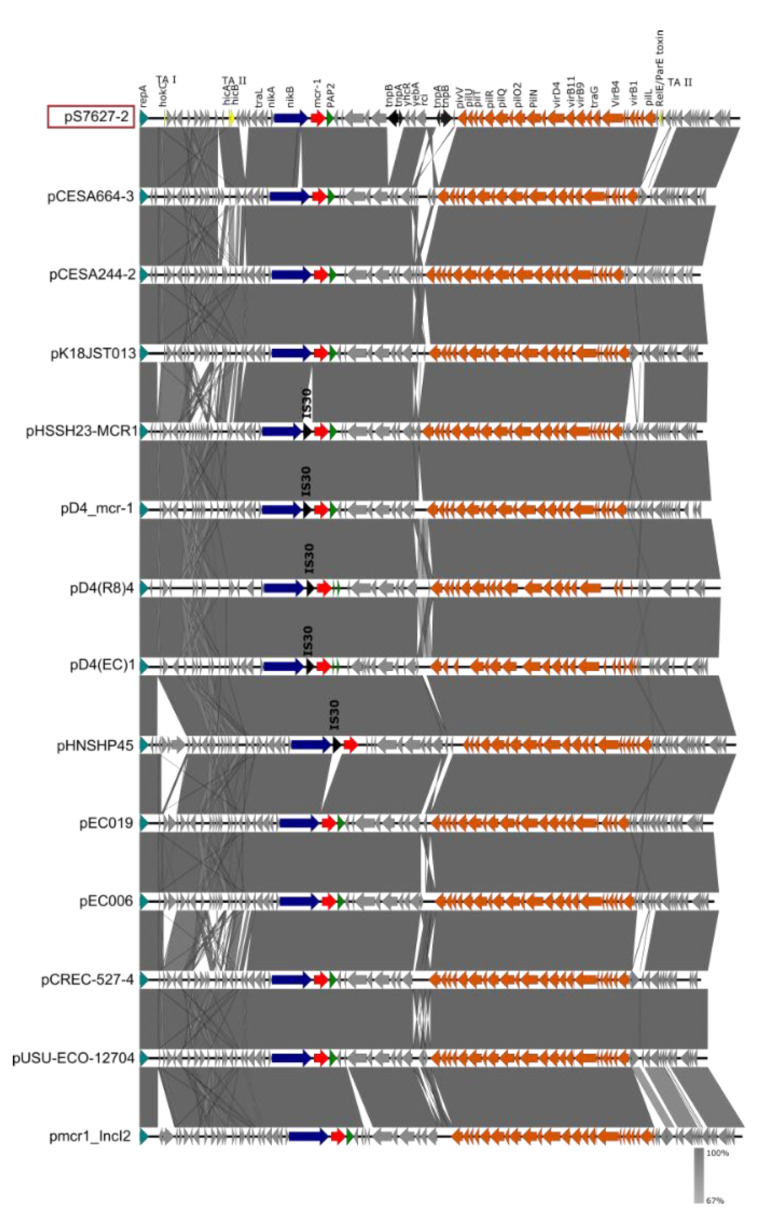
Comparison of the IncI2 plasmid of the pS7627-2 isolate carrying the *mcr-1.1* gene to the IncI2 plasmids of *Salmonella* (pCESA664-3, pCESA244-2, pK18JST013, pHSSH23-MCR1, pD4_mcr-1, pD4(R8)4, pD4(EC)1) and *E. coli* (pHNSHP45, pEC019, pEC006, pCREC-527-4, pUSU-ECO-12704, pmcr1_IncI2) isolates. Arrows represent the position of open reading frames. The GenBank accession numbers for these plasmids are listed in Supplementary Table S1. Regions of >99% identity are indicated in dark grey in the bar between compared plasmid sequences. The *RepA* gene is colored turquoise, genes associated with the plasmid backbone are colored orange, antibiotic resistance genes are colored red, the *PAP2* gene is colored green, insertion sequences are colored black, genes encoding toxin–antitoxin (TA) systems of type I (TA-I) and type II (TA-II) are colored yellow, and other genes are colored gray.

A BLASTN comparison of the complete sequence of plasmid IncI2 to the NCBI GenBank database revealed similar plasmids in *E. coli*, *S*. Typhimurium, and *K. aerogenes* isolates with an identity of 99% and a query cover from 97–56%. No further isolates of the serotype *S*. Bovismorbificans carrying similar plasmids were identified. Comparisons with previously published *Salmonella* and *E. coli* IncI2 plasmids (Supplementary Table S1), carrying *mcr-1.1* gene, are shown in Figure 2. The IncI2 plasmid pS7627-2 has a similar plasmid backbone and a similar genetic environment surrounding the *mcr-1.1* gene (*nikB*-*mcr-1.1*-*PAP*) compared to other IncI2 plasmids (pCESA664-3, pCESA244-2, pK18JST013) previously isolated in China and Korea [33]. The IS*Apl1* insertion sequence belonging to the IS*30* family was not identified near *mcr-1.1*.

#### 3.3.3. Monophasic Variant of Salmonella Typhimurium 4,5:i:- (SLR1_8094)

**Figure 3:**
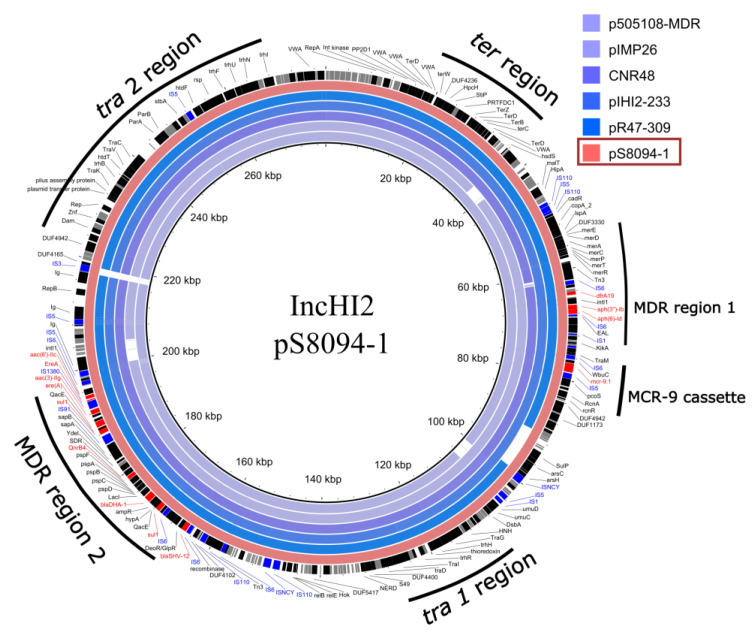
Circular map of the BLASTN comparison of IncHI2 type plasmids pS80904-1 (*S*. Typhimurium), pR47-309 (*C. freundii*), pHI2-233 (*E. hormaechei*), CNR48 (*K. pneumoniae*), pIMP26 (*E. cloacae*), and p505108-MDR (*C. sakazakii*) generated by the BLAST Ring Image Generator tool. The pS80904-1 plasmid was used as a reference. The outer circle denotes the annotation of the reference plasmid. The AMR genes are highlighted by red boxes, genes encoding hypothetical proteins highlighted by grey boxes, IS-elements highlighted by blue boxes, and other genes highlighted by black boxes. The backbone (tra1 and tra2 region), two accessory resistance regions (MDR region 1, MDR region 2), and *mcr*-9 cassette (*rcnR*-*rcnA*-*pcoE*-*pcoS*-IS*5*-*mcr-9*-*wbuC*-IS*6*) are indicated by black curves.

The MDR colistin-susceptible, ESBL-producing *S.* Typhimurium 4,5:i:- isolate SLR1_8094 had only the 278 kb conjugative plasmid pS8094-1 of the type IncHI2 (Table 3). Comparison of the full plasmid sequence of pS8094-1 against the NCBI GenBank database showed that it has a high similarity and query coverage to other IncHI2 plasmids from bacteria of non-*Salmonella* origin, e.g., *E. hormaechei* pHI2-233 (97% coverage and 99% sequence identity; CP049047.1); *K. pneumoniae* pCNR48 (97% coverage and 99% sequence identity; LT994835.1); *C. sakazakii* p505108-MDR (97% coverage and 99% sequence identity; KY978628 [34]); *E. cloacae* pIMP26 (95% coverage and 99% sequence identity; MH399264.1); and *C. freundii* pR47-309 (95% coverage and 99% sequence identity; CP040696.1 [35]). Similar plasmids in *Salmonella* had 99% sequence identity but relatively low query coverage: pSA20094620.1 (83% coverage; CP030186) and pXXB1403 (81% coverage; CP059887.1). The plasmid pS8094-1 possesses the core backbone markers of typical IncHI2 plasmids, including genes encoding replication initiation, *parAB* and *parMR* for partition, the *tra1* and *tra2* regions for conjugal transfer, and the tellurite resistance region (*terY3Y2XY1WZABCDEF*) (Figure 3) [36].

The phenotypic resistance to the different classes of antimicrobial agents, as well as the production of ESBL correlated with the identified AMR genes on the IncHI2 plasmid and chromosome (Table 2). The gene responsible for tetracycline resistance *tet(B)* was located on the chromosome (Table 3). The IncHI2 plasmid carried genes responsible for resistance to aminoglycosides (*aac(3′’)-IIg*, *aac(6’)-IIc*, *aph(6)-Id*, *aph(3’’)-Ib*), sulphonamide and trimethoprim (*sul1* x2, *dfrA19*), quinolone (*qnrB4*), macrolides (*ere(A)*), and β-lactam antibiotics (*blaDHA-1*, *blaSHV-12*). These AMR genes were located in two different MDR regions (MDR region 1 and MDR region 2) on the IncHI2 plasmid (Figure 3). The identified *mcr* cassette contained an *mcr-9* gene located near the MDR region 1. The genetic environment surrounding *mcr-9* was *rcnR*-*rcnA*-*pcoE*-*pcoS*-IS*5*-*mcr-9*-*wbuC* with the IS*6* element, which relates to the *mcr-9* cassette type on plasmid p17277A_477 (IncHI2) according to the classification suggested by Li et al., 2020 [37]. This *mcr* cassette lacks the downstream regulatory genes (*qseC* and *qseB*), which were proposed to be involved in the induction of the colistin resistance mediated by *mcr-9* [37].

## 4. Discussion

The novelty of our study is the detection and description of four Russian NTS isolates at a genomic level, revealing details about plasmids carrying *mcr* genes. Three isolates belong to *S*. Enteritidis (*n* = 2) and *S*. Bovismobificans (*n* = 1), and all of them were characterized by the presence of an *mcr-1.1* gene and had phenotypical resistance to colistin. Colistin is one of the so-called “last-resort” antimicrobials used to treat MDR infections caused by members of the Enterobacteriaceae family. The fourth isolate belonged to monophasic *S*. Typhimurium and, while carrying an *mcr-9* gene, was susceptible to colistin. The successful combination of the genome sequencing approaches based on short and long reads allowed us to accurately reconstruct the complete sequences of the chromosomes and plasmids for each isolate, permitting detailed analyses of the plasmid’s structure that carries the *mcr* genes. To the best of our knowledge, this is the first study that describes cases of NTS strain isolation with *mcr-*mediated resistance to colistin in Russia with a detailed analysis of both AMR genes and phenotypical resistances.

Based on the data obtained, we conclude that the prevalence rate of *mcr-1.1* in *Salmonella* isolates is very low (0.5%), which correlates well with previous observations [38,39]. Nevertheless, three of the studied isolates were phenotypically resistant to at least three classes of antimicrobial drugs. These MDR phenotypes were accurately confirmed by the presence of corresponding AMR genes in the genomes, corroborating the predictive power of WGS [40]. Based on the WGS data, we show that the sequenced isolates are genetically unique by core genome features (SNP and alleles of core genes) among all available genomes sequenced to date for *Salmonella*. Moreover, we did not find any similarity to previously isolated *S*. Enteritidis representatives from the outbreaks and sporadic cases circulating in Russia.

Based on the analyzed genomic sequences, we found that the *mcr-1.1* genes of two *S*. Enteridis isolates were localized on IncX4 plasmids, whilst in the *S*. Bovismorbificans isolate the *mcr-1.1* was on the IncI2 plasmid. An *mcr-9* gene variant was detected on the IncHI2 plasmid in the monophasic human isolate of *S*. Typhimurium. The data obtained are consistent with previous observations, where it was shown that plasmid types predominantly harboring *mcr* genes are IncX4, IncI2, and IncHI2 [39,41,42]. Interestingly, only the IncHI2 plasmid was associated with additional antibiotic resistance genes, while the IncX4 and IncI2 plasmids carried only one *mcr* gene. The absence of a flanking IS*Apl1* near the *mcr* cassettes on the IncX4 and IncI2 plasmids suggest loss of the IS*Apl1* elements after the integration of the transposon. It further suggests that the loss of IS*Apl1* anchors the *mcr-1* gene on the plasmid, which probably indicates the stability of the *mcr* genes on plasmids [6].

By comparing the plasmid sequences with previously published data, we found that the IncX4 plasmids in the two *S*. Enteritidis strains in this study showed high levels of similarity to an epidemic pCSZ4-like IncX4 plasmid [30]. The IncX4 epidemic plasmid pCSZ4 contains an identical *mcr* cassette with absence of the IS*Apl1* transposon. It should be noted that IncX4 is one of the most widespread plasmid types among *E. coli* and other *Enterobacteriaceae.* This plasmid is characterized by a high frequency of self-transfer between different species of *Enterobacteriaceae,* indicating its high epidemiological significance [43]. It was also interesting to note that *mcr-1.1* positive isolates were found among the *Salmonella* isolates belonging to the serotype Enteritidis, which is the dominant etiological serotype for morbidity worldwide [44,45]. In the present study, *S*. Enteritidis were isolated during an outbreak of salmonellosis and from a poultry product, which indicates the opportunity for successful circulation of epidemic pCSZ4-like IncX4 plasmids in *S*. Enteritidis populations. To date, there are only a few cases of *S*. Enteritidis described with plasmids carrying *mcr* genes, but none of these studies detected the epidemic IncX4 plasmid in *S*. Enteritidis isolates [38,46].

Remarkably, the isolate carrying the *mcr-9* gene (monophasic *S*.Typhimurium) did not show phenotypical resistance to colistin. According to earlier studies [47,48], this phenomenon was previously observed, although colistin-resistant *Salmonella enterica* carrying the *mcr-9* gene was also discovered [49]. Obviously, *mcr-9* is widely distributed among various *Enterobacteriaceae* species, and the most common *mcr-9* carrying plasmid is shown to be IncHI2 [37]. Moreover, co-occurrence of the *mcr-9* gene with various antibiotic resistance genes was shown, indicating the epidemiological significance of IncHI2 plasmids in spreading of AMR genes [50]. Most often, and consistent with our data, the structure of the *mcr-9* cassettes in *Salmonella* plasmids presents as IS*5*-*mcr-9*-*wbuC*-IS*6* and lacks the downstream regulatory genes *qseB* and *qseC* [48,50].

The data provided here expand the global picture fordissemination of mobilized colistin resistance in *Enterobacteriaceae*. However, future retrospective studies from different sources over a longer period of time are urgently needed to assess the prevalence of plasmid-mediated colistin resistance among NTS strains in Russia.

## Figures and Tables

**Table 1:** Russian isolates characterized by presence of *mcr* genes.

	Serotype	Isolation Date (mm/yyyy)	Country:City	Epidemiological Background	Source	*mcr*-Type
SLR1_8250	*S.* Enteritidis	04/2019	Russia:Yakutiya	ND	food (chicken meat)	1
SLR1_8245	*S.* Enteritidis	07/2019	Russia:El’ban	Outbreak	human	1
SLR1_7627	*S.* Bovismorbificans	09/2018	Russia:Irkutsk	Sporadic	human	1
SLR1_8094	*S.* Typhimurium monophasic variant [4,5:i:-]	06/2019	Russia:Ulan-Ude	Sporadic	human	9

ND—data not available.

**Table 2:** Antimicrobial susceptibility testing of four *Salmonella* isolates containing an *mcr* gene based on MIC and AMR genes associated with resistance to particular antimicrobial agents.

Antimicrobial Class	Antimicrobial Agent	SLR1_8250		SLR1_7627		SLR1_8245		SLR1_8094	
polymyxins	colistin	8 (R)	mcr-1.1	8 (R)	mcr-1.1	8 (R)	mcr-1.1	1 (S)	mcr-9
penicillins	ampicillin	2 (S)		>128 (R)	*blaTEM-1B*	2 (S)		>128 (R)	*blaDHA-1, blaSHV-12*
	ampicillin-sulbactam	2/1 (S)		32/8 (R)		2/1 (S)		64/32 (R)	
	piperacillin	2 (S)		>128 (R)		2 (S)		>128 (R)	
	piperacillin-tazobactam	≤2/4 (S)		≤1/4 (S)		2/4 (S)		2/4 (S)	
cephems	cefotaxime	0.12 (S)		0.12 (S)		0.12 (S)		>8 (R)	*blaDHA-1, blaSHV-12*
	ceftazidime	0.5 (S)		0.25 (S)		0.5 (S)		>16 (R)	
	cefepime	≤0.12 (S)		≤0.12 (S)		≤0.12 (S)		2 (S)	
monobactams	aztreonam	≤0.12 (S)		≤0.12 (S)		≤0.12 (S)		>16 (R)	*blaDHA-1, blaSHV-12*
carbapenems	meropenem	≤0.12 (S)		≤0.12 (S)		≤0.12 (S)		≤0.12 (S)	
	ertapenem	≤0.015 (S)		≤0.015 (S)		≤0.015 (S)		0.03 (S)	
aminoglycosides	gentamicin	0.5 (S)		0.5 (S)	*aph(3’’)-Ib, aph(6)-Id,* *aadA1*	0.5 (S)		>32 (R)	*aac(3)-II, aac(6’)-IIc, aph(6)-Id, aph(3’’)-Ib*
	amikacin	≤1 (S)		2 (S)		≤1 (S)		2(S)	
	tobramycin	1 (S)		1 (S)		0.5 (S)		>8 (R)	
trimethoprim-sulfonamide	trimethoprim-sulfamethoxazole	0.06/1.19 (S)		>4/76 (R)	*sul1, sul2, dfrA1*	0.06/1.19 (S)		>4/76 (R)	*sul1, dfrA19*
quinolones	ciprofloxacin	≤0.06 (S)		≤0.06 (S)		≤0.06 (S)		0.25 (R)	*qnrB4*
amphenicols	chloramphenicol	8 (S)		4 (S)		>32 (R)	*catA1*	4 (S)	
tetracyclines	tetracycline	2 (S)		>32 (R)	*tet(A)*	>32 (R)	*tet(A)*	>32 (R)	*tet(B)*

S—Susceptible; R—Resistant.

**Table 3:** Genome content of completed genomes of Russian Salmonella isolates.

Serotype, Strain Name, NCBI GenBank acc.	Replicon Name	Size, bp	AMR Genes ^1^	Replion Type(s) ^2^	Mobility Prediction for the Plasmid ^3^
*S*. Enteritidis, SLR1_8250, CP060522-CP060525	chromosome	4,679,617	ND	chromosome	ND
	pS8250-1	59,372	ND	IncFII(S), IncFIB(S)	non-mobilizable
	pS8250-2	33,310	*mcr-1.1*	IncX4	conjugative
	pS8250-3	2096	ND	ColpVC	mobilizable
*S*. Enteritidis, SLR1_8245	chromosome	4,680,323	ND	chromosome	-
	pS8245-1	59,372	ND	IncFII(S), IncFIB(S)	non-mobilizable
	pS8245-2	56,813	*catA1, tet(A)*	IncX1	conjugative
	pS8245-3	33,310	*mcr-1.1*	IncX4	conjugative
*S*. Bovismorbificans, SLR1_7627, CP060517-CP060521	chromosome	4,715,485	ND	chromosome	ND
	pS7627-1	233,305	*aph(3’’)-Ib, aph(6)-Id, aadA1, dfrA1, tet(A), blaTEM-1B, sul1, sul2*	IncHI2, IncQ1	non-mobilizable
	pS7627-2	64,443	*mcr-1.1*	IncI2	conjugative
	pS7627-3	4073	ND	rep_cluster_2350 ^3^	mobilizable
	pS7627-4	3830	ND	rep_cluster_2335 ^3^	mobilizable
*S*. Typhimurium [4,5:i:-], SLR1_8094, CP060515-CP060516	chromosome	5,017,156	*tet(B)*	chromosome	ND
	pS8094-1	278,034	*aph(3’’)-Ib, aph(6)-Id, aac(3)-IIg, aac(6’)-IIc, ere(A), qnrB4, dfrA19,mcr-9, blaSHV-12, blaDHA-1, sul1* x2	IncHI2	conjugative

ND—not detected; ^1^ Predicted by ResFinder; ^2^ Predicted by PlasmidFinder; ^3^ Predicted by MOB-suite.

## Data Availability

Raw sequencing reads of strains as well as complete genome assemblies have been deposited in the NCBI BioProject database under accession numbers PRJNA484865 (SAMN15666299 = SLR1_8250, SAMN15666300 = SLR1_7627, SAMN15666301 = SLR1_8094, SAMN20345806 = SLR1_8245, SAMN20345807 = SLR1_8239).

## Supplementary Materials

The following are available online at https://www.mdpi.com/article/10.3390/microorganisms9122515/s1. Table S1: Detailed information of IncX4, IncI2 plasmids used for comparative analysis. Figures S1–S4: Defining the uniqueness of isolates using the NCBI Pathogen Detection system and EnteroBase.

## References

[1] (2013). Centers for Disease Control and Prevention. National Salmonella Surveillance Annual Report, 2011. Atlanta, Georgia: US Department of Health and Human Services, CDC. https://www.cdc.gov/ncezid/dfwed/PDFs/salmonella-annual-report-2011-508c.pdf.

[2] Majowicz S.E., Musto J., Scallan E., Angulo F.J., Kirk M., O’Brien S.J., Jones T.F., Fazil A., Hoekstra R.M., International Collaboration on Enteric Disease “Burden of Illness” Studies (2010). The global burden of nontyphoidal Salmonella gastroenteritis. Clin. Infect. Dis..

[3] State Report on the State of Sanitary and Epidemiological Well-Being of the Population in the Russian Federation in 2020. https://www.rospotrebnadzor.ru/bitrix/redirect.php?event1=file&event2=download&event3=gd-seb_02.06-_s-podpisyu_.pdf&goto=/upload/iblock/5fa/gd-seb_02.06-_s-podpisyu_.pdf.

[4] Rozhnova S.S., Kuleshov K.V., Pavlova A.S., Guseva A.N., Kozhakhmetova T.A., Akulova N.K., Podkolzin A.T. (2020). Heterogeneity of Salmonella isolates obtained from various sources in Russian Federation 2010–2019. Epidemiol. Infect. Dis..

[5] Caniaux I., van Belkum A., Zambardi G., Poirel L., Gros M.F. (2017). MCR: Modern colistin resistance. Eur. J. Clin. Microbiol. Infect. Dis..

[6] Wang R., van Dorp L., Shaw L.P., Bradley P., Wang Q., Wang X., Jin L., Zhang Q., Liu Y., Rieux A. (2018). The global distribution and spread of the mobilized colistin resistance gene mcr-1. Nat. Commun..

[7] Lima T., Domingues S., Da Silva G.J. (2019). Plasmid-Mediated Colistin Resistance in Salmonella enterica: A Review. Microorganisms.

[8] Scott H.M., Acuff G., Bergeron G., Bourassa M.W., Gill J., Graham D.W., Kahn L.H., Morley P.S., Salois M.J., Simjee S. (2019). Critically important antibiotics: Criteria and approaches for measuring and reducing their use in food animal agriculture. Ann. N. Y. Acad. Sci..

[9] Liu Y.Y., Wang Y., Walsh T.R., Yi L.X., Zhang R., Spencer J., Doi Y., Tian G.B., Dong B.L., Huang X.H. (2016). Emergence of plasmid-mediated colistin resistance mechanism MCR-1 in animals and human beings in China: A microbiological and molecular biological study. Lancet Infect. Dis..

[10] Poirel L., Jayol A., Nordmann P. (2017). Polymyxins: Antibacterial Activity, Susceptibility Testing, and Resistance Mechanisms Encoded by Plasmids or Chromosomes. Clin. Microbiol. Rev..

[11] Vallejos-Sanchez K., Tataje-Lavanda L., Villanueva-Perez D., Bendezu J., Montalvan A., Zimic-Peralta M., Fernandez-Sanchez M., Fernandez-Diaz M. (2019). Whole-Genome Sequencing of a Salmonella enterica subsp. enterica Serovar Infantis Strain Isolated from Broiler Chicken in Peru. Microbiol. Resour. Announc..

[12] Rozwandowicz M., Brouwer M.S.M., Fischer J., Wagenaar J.A., Gonzalez-Zorn B., Guerra B., Mevius D.J., Hordijk J. (2018). Plasmids carrying antimicrobial resistance genes in Enterobacteriaceae. J. Antimicrob. Chemother..

[13] Datta N., Hughes V.M. (1983). Plasmids of the same Inc groups in Enterobacteria before and after the medical use of antibiotics. Nature.

[14] Matamoros S., van Hattem J.M., Arcilla M.S., Willemse N., Melles D.C., Penders J., Vinh T.N., Hoa N.T., Bootsma M.C.J., van Genderen P.J. (2017). Global phylogenetic analysis of Escherichia coli and plasmids carrying the mcr-1 gene indicates bacterial diversity but plasmid restriction. Sci. Rep..

[15] Doumith M., Godbole G., Ashton P., Larkin L., Dallman T., Day M., Day M., Muller-Pebody B., Ellington M.J., de Pinna E. (2016). Detection of the plasmid-mediated mcr-1 gene conferring colistin resistance in human and food isolates of Salmonella enterica and Escherichia coli in England and Wales. J. Antimicrob. Chemother..

[16] Manageiro V., Clemente L., Romao R., Silva C., Vieira L., Ferreira E., Canica M. (2019). IncX4 Plasmid Carrying the New mcr-1.9 Gene Variant in a CTX-M-8-Producing Escherichia coli Isolate Recovered from Swine. Front. Microbiol..

[17] Ye H., Li Y., Li Z., Gao R., Zhang H., Wen R., Gao G.F., Hu Q., Feng Y. (2016). Diversified mcr-1-Harbouring Plasmid Reservoirs Confer Resistance to Colistin in Human Gut Microbiota. MBio.

[18] European Committee on Antimicrobial Susceptibility Testing. Breakpoint Tables for Interpretation of MICs and Zone Di-ameters. Version 11.0, valid from 1 January 2021. https://www.eucast.org/fileadmin/src/media/PDFs/EUCAST_files/Breakpoint_tables/v_11.0_Breakpoint_Tables.pdf.

[19] EUCAST Guidelines for Detection of Resistance Mechanisms and Specific Resistances of Clinical and/or Epidemiological Importance. Version 2.0. https://www.eucast.org/fileadmin/src/media/PDFs/EUCAST_files/Resistance_mechanisms/EUCAST_detection_of_resistance_mechanisms_170711.pdf.

[20] Wick R.R., Judd L.M., Gorrie C.L., Holt K.E. (2017). Unicycler: Resolving bacterial genome assemblies from short and long sequencing reads. PLoS Comput. Biol..

[21] Koren S., Walenz B.P., Berlin K., Miller J.R., Bergman N.H., Phillippy A.M. (2017). Canu: Scalable and accurate long-read assembly via adaptive k-mer weighting and repeat separation. Genome Res..

[22] Seibt K.M., Schmidt T., Heitkam T. (2018). FlexiDot: Highly customizable, ambiguity-aware dotplots for visual sequence analyses. Bioinformatics.

[23] Achtman M., Wain J., Weill F.X., Nair S., Zhou Z.M., Sangal V., Krauland M.G., Hale J.L., Harbottle H., Uesbeck A. (2012). Multilocus Sequence Typing as a Replacement for Serotyping in Salmonella enterica. PLoS Pathog..

[24] Robertson J., Nash J.H.E. (2018). MOB-suite: Software tools for clustering, reconstruction and typing of plasmids from draft assemblies. Microb. Genom..

[25] Carattoli A., Zankari E., Garcia-Fernandez A., Larsen M.V., Lund O., Villa L., Aarestrup F.M., Hasman H. (2014). In Silico Detection and Typing of Plasmids Using PlasmidFinder and Plasmid Multilocus Sequence Typing. Antimicrob. Agents Chemother..

[26] Bortolaia V., Kaas R.S., Ruppe E., Roberts M.C., Schwarz S., Cattoir V., Philippon A., Allesoe R.L., Rebelo A.R., Florensa A.F. (2020). ResFinder 4.0 for predictions of phenotypes from genotypes. J. Antimicrob. Chemother..

[27] Feldgarden M., Brover V., Gonzalez-Escalona N., Frye J.G., Haendiges J., Haft D.H., Hoffmann M., Pettengill J.B., Prasad A.B., Tillman G.E. (2021). AMRFinderPlus and the Reference Gene Catalog facilitate examination of the genomic links among antimicrobial resistance, stress response, and virulence. Sci. Rep..

[28] Sullivan M.J., Petty N.K., Beatson S.A. (2011). Easyfig: A genome comparison visualizer. Bioinformatics.

[29] Alikhan N.F., Petty N.K., Zakour N.L.B., Beatson S.A. (2011). BLAST Ring Image Generator (BRIG): Simple prokaryote genome comparisons. BMC Genom..

[30] Sun J., Fang L.X., Wu Z., Deng H., Yang R.S., Li X.P., Li S.M., Liao X.P., Feng Y., Liu Y.H. (2017). Genetic Analysis of the IncX4 Plasmids: Implications for a Unique Pattern in the mcr-1 Acquisition. Sci. Rep..

[31] Hu Y., Nguyen S.V., Wang W., Gan X., Dong Y., Liu C., Cui X., Xu J., Li F., Fanning S. (2021). Antimicrobial Resistance and Genomic Characterization of Two mcr-1-Harboring Foodborne Salmonella Isolates Recovered in China, 2016. Front. Microbiol..

[32] Tang B., Chang J., Zhang L., Liu L., Xia X., Hassan B.H., Jia X., Yang H., Feng Y. (2020). Carriage of Distinct mcr-1-Harboring Plasmids by Unusual Serotypes of Salmonella. Adv. Biosyst..

[33] Moon D.C., Kim S.J., Mechesso A.F., Kang H.Y., Song H.J., Choi J.H., Yoon S.S., Lim S.K. (2021). Mobile Colistin Resistance Gene mcr-1 Detected on an IncI2 Plasmid in Salmonella Typhimurium Sequence Type 19 from a Healthy Pig in South Korea. Microorganisms.

[34] Shi L., Liang Q., Zhan Z., Feng J., Zhao Y., Chen Y., Huang M., Tong Y., Wu W., Chen W. (2018). Co-occurrence of 3 different resistance plasmids in a multi-drug resistant Cronobacter sakazakii isolate causing neonatal infections. Virulence.

[35] Zhou W., Chen Q., Qian C., Shen K., Zhu X., Zhou D., Lu W., Sun Z., Liu H., Li K. (2019). In Vitro Susceptibility and Florfenicol Resistance in Citrobacter Isolates and Whole-Genome Analysis of Multidrug-Resistant Citrobacter freundii. Int. J. Genom..

[36] Johnson T.J., Wannemeuhler Y.M., Scaccianoce J.A., Johnson S.J., Nolan L.K. (2006). Complete DNA sequence, comparative genomics, and prevalence of an IncHI2 plasmid occurring among extraintestinal pathogenic Escherichia coli isolates. Antimicrob. Agents Chemother..

[37] Li Y., Dai X., Zeng J., Gao Y., Zhang Z., Zhang L. (2020). Characterization of the global distribution and diversified plasmid reservoirs of the colistin resistance gene mcr-9. Sci. Rep..

[38] Cui M., Zhang J., Gu Z., Li R., Chan E.W., Yan M., Wu C., Xu X., Chen S. (2017). Prevalence and Molecular Characterization of mcr-1-Positive Salmonella Strains Recovered from Clinical Specimens in China. Antimicrob. Agents Chemother..

[39] Sia C.M., Greig D.R., Day M., Hartman H., Painset A., Doumith M., Meunier D., Jenkins C., Chattaway M.A., Hopkins K.L. (2020). The characterization of mobile colistin resistance (mcr) genes among 33,000 Salmonella enterica genomes from routine public health surveillance in England. Microb. Genom..

[40] Cooper A.L., Low A.J., Koziol A.G., Thomas M.C., Leclair D., Tamber S., Wong A., Blais B.W., Carrillo C.D. (2020). Systematic Evaluation of Whole Genome Sequence-Based Predictions of Salmonella Serotype and Antimicrobial Resistance. Front. Microbiol..

[41] Migura-Garcia L., Gonzalez-Lopez J.J., Martinez-Urtaza J., Sanchez J.R.A., Moreno-Mingorance A., Rozas A.P.d., Hofle U., Ramiro Y., Gonzalez-Escalona N. (2019). mcr-Colistin Resistance Genes Mobilized by IncX4, IncHI2, and IncI2 Plasmids in Escherichia coli of Pigs and White Stork in Spain. Front. Microbiol..

[42] Wu R., Yi L.X., Yu L.F., Wang J., Liu Y., Chen X., Lv L., Yang J., Liu J.H. (2018). Fitness Advantage of mcr-1-Bearing IncI2 and IncX4 Plasmids In Vitro. Front. Microbiol..

[43] Lo W.U., Chow K.H., Law P.Y., Ng K.Y., Cheung Y.Y., Lai E.L., Ho P.L. (2014). Highly conjugative IncX4 plasmids carrying blaCTX-M in Escherichia coli from humans and food animals. J. Med. Microbiol..

[44] Rodrigue D.C., Tauxe R.V., Rowe B. (1990). International increase in Salmonella enteritidis: A new pandemic?. Epidemiol. Infect..

[45] Christenson J.C. (2013). Salmonella infections. Pediatr. Rev..

[46] Lu X., Zeng M., Xu J., Zhou H., Gu B., Li Z., Jin H., Wang X., Zhang W., Hu Y. (2019). Epidemiologic and genomic insights on mcr-1-harbouring Salmonella from diarrhoeal outpatients in Shanghai, China, 2006–2016. EBioMedicine.

[47] Carroll L.M., Gaballa A., Guldimann C., Sullivan G., Henderson L.O., Wiedmann M. (2019). Identification of Novel Mobilized Colistin Resistance Gene mcr-9 in a Multidrug-Resistant, Colistin-Susceptible Salmonella enterica Serotype Typhimurium Isolate. MBio.

[48] Tyson G.H., Li C., Hsu C.H., Ayers S., Borenstein S., Mukherjee S., Tran T.T., McDermott P.F., Zhao S. (2020). The mcr-9 Gene of Salmonella and Escherichia coli Is Not Associated with Colistin Resistance in the United States. Antimicrob. Agents Chemother..

[49] Cha M.H., Woo G.J., Lee W., Kim S.H., Woo J.H., Kim J., Ryu J.G., Kwak H.S., Chi Y.M. (2020). Emergence of Transferable mcr-9 Gene-Carrying Colistin-Resistant Salmonella enterica Dessau ST14 Isolated from Retail Chicken Meat in Korea. Foodborne Pathog. Dis..

[50] Wang Y., Li Z., Lyu N., Ma S., Liu F., Hu Y., Gao G.F., Zhu B. (2021). Comparative genomic analysis of mobile colistin resistance gene mcr-9 in Salmonella enterica. J. Infect..

