# Supplementary material for: Mobile Colistin Resistance Genetic Determinants of Non-Typhoid Salmonella enterica Isolates from Russia
Source: Microorganisms. 2021 Dec 4;9(12):2515. doi: 10.3390/microorganisms9122515 (PMC8705591; doi:10.3390/microorganisms9122515)
Supplement: Supplementary file 1 [file microorganisms-09-02515-s001.zip › Figures S1-S4_proof_kv.pdf]

Defining the uniqueness of isolates using the NCBI Pathogen Detection system

Using the NCBI Pathogen Detection system a request for the isolates under study can be submitted to the database. The screenshots shown below display the results for the four Russian isolates described in our study (available under URL link https://www.ncbi.nlm.nih.gov/pathogens/isolates/#SLR1_8250%20OR%20SLR1_7627%20OR%20SLR1_8245%20OR%20SLR1_8094). To identify the relatedness of the studied isolates to other isolates in the NCBI Pathogen Detection system, a 50-SNP single-linkage clustering was automatically created (Figure S1). When the "SNP cluster" field remains empty, it indicates that the system did not find isolates in the database with $\leq$50 SNP differences. For the isolates in our study, i.e. SLR1_7627, SLR1_8094, and SLR1_8250 no closely related isolates were found in the databases. However, isolates SLR1_8245 and SLR1_8239 were similar (0 SNP differences) and they formed a SNP cluster named PDS000093458.2 (Figure S2). These two isolates SLR1_8245 and SLR1_8239 belonged to the same salmonellosis outbreak (El'ban village in the Khabarovsk Territory) (Figure S2). So, we concluded that our isolates did not have a close relationship (at the $\leq$ 50 SNP threshold) to other isolates in the NCBI Pathogen Detection system or to each other – with the exception of isolates SLR1_8245 and SLR1_8239.

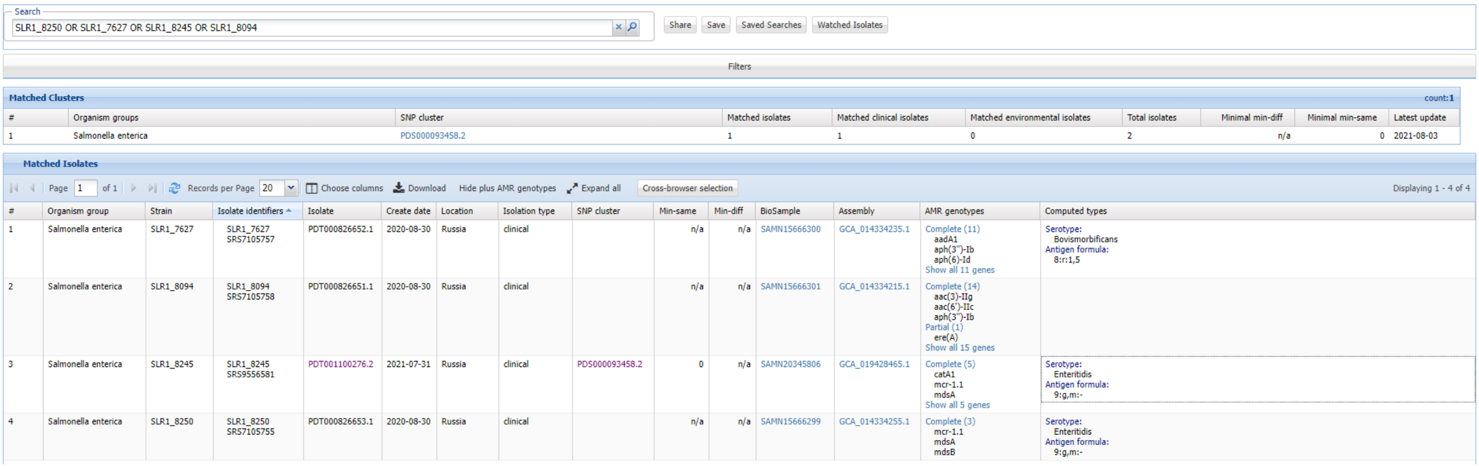

Figure S1. Results of a similarity check (threshold $\leq$ 50 SNPs) of our analysed isolates with isolates contained in the NCBI Pathogen Detection system using URL https://www.ncbi.nlm.nih.gov/pathogens/isolates/#SLR1_8250%20OR%20SLR1_7627%20OR%20SLR1_8245%20OR%20SLR1_8094

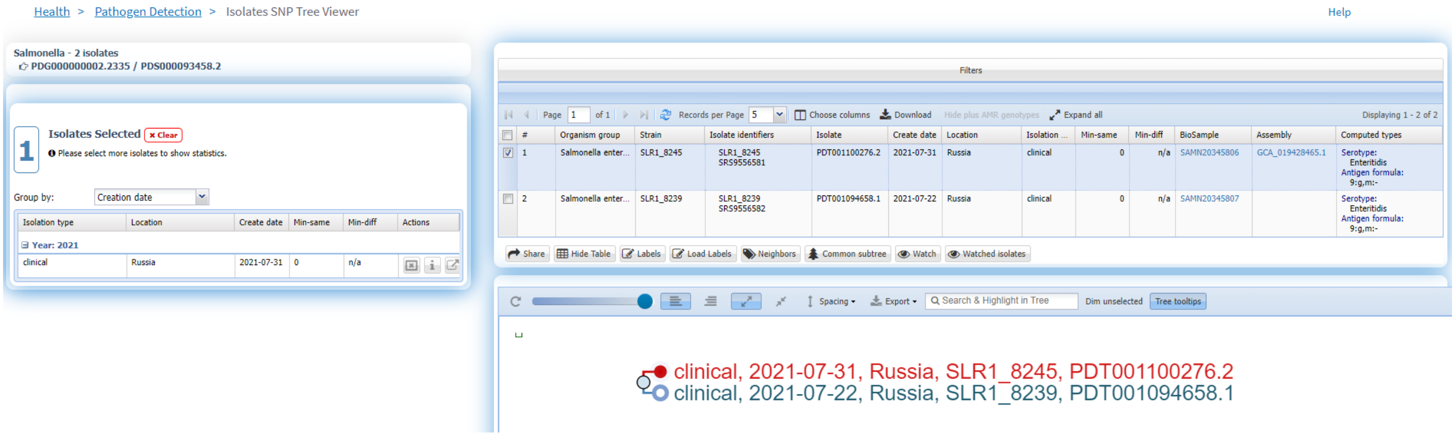

Figure S2. Screenshot of SNP cluster PDS000093458.2. Isolates *S.* Enteritidis SLR1_8245 and SLR1_8239 were isolated from the same outbreak and have 0 SNP differences.

Definition of uniqueness of isolates using EnteroBase system.

For each of the isolates EnteroBase system automatically calculated cluster numbers based on algorithms of hierarchical clustering where HC0 (indistinguishable), HC2 (two loci differences), HC5 (five loci differences), HC10 (differ in 10 loci), and HC20 (differ in 20 loci) etc. (Figure S3). For isolates SLR1_7627 (HC0_238586), SLR1_8250 (HC0_238575), SLR1_8245 (HC0_278706) HC0 cluster number was the same at levels HC2, HC5, HC10, and HC20, and for isolate SLR1_8094 HC0_236467 was the same at levels HC2, HC5, HC10.

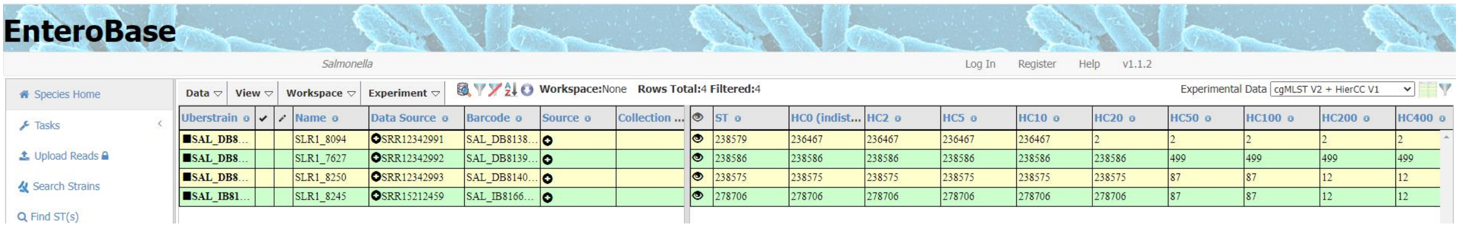

Uberstrain	✔	✎	Name	Data Source	Barcode	Source	Collection ...		ST	HC0 (indist...	HC2	HC5	HC10	HC20	HC50	HC100	HC200	HC400
SAL_DB8...			SLR1_8094	SRR12342991	SAL_DB8138...				238579	236467	236467	236467	236467	2	2	2	2	2
SAL_DB8...			SLR1_7627	SRR12342992	SAL_DB8139...				238586	238586	238586	238586	238586	238586	499	499	499	499
SAL_DB8...			SLR1_8250	SRR12342993	SAL_DB8140...				238575	238575	238575	238575	238575	238575	87	87	12	12
SAL_IB81...			SLR1_8245	SRR15212459	SAL_IB8166...				278706	278706	278706	278706	278706	278706	87	87	12	12

Figure S3. Studied isolates and their sequence types (ST) and cluster numbers (HC0, HC2, HC5, HC10, HC20 etc.) calculated based on algorithms of hierarchical clustering of cgMLST data in the EnteroBase system.

To show that the database did not contain any isolate that was identical to our isolates (at least with a difference of 10 loci) we performed separate searching of values of HC10 cluster numbers of each studied isolate in EnteroBase. The example of the query

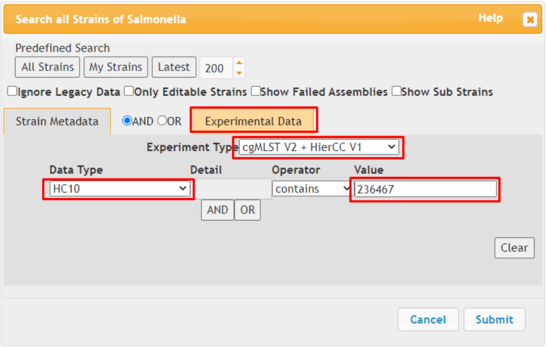

For each HC10 value we received only one isolate (HC10_238586 - SLR1_7627, HC10_236467 - SLR1_8094, and HC10_238575 - SLR1_8250) (Figure S4).

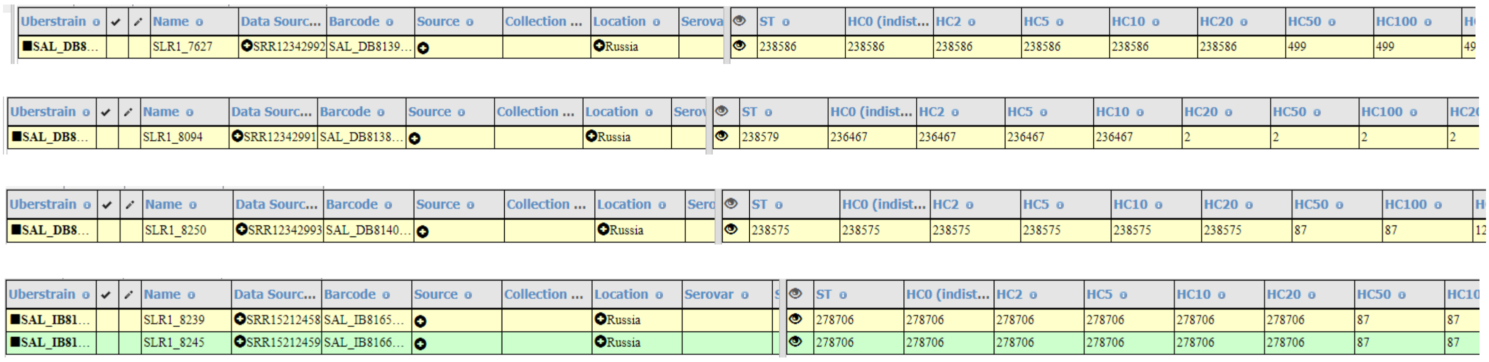

Uberstrain	✔	✎	Name	Data Sourc...	Barcode	Source	Collection ...	Location	Serova		ST	HC0 (indist...	HC2	HC5	HC10	HC20	HC50	HC100	H
SAL_DB8...			SLR1_7627	SRR12342992	SAL_DB8139...			Russia			238586	238586	238586	238586	238586	238586	499	499	49

Uberstrain	✔	✎	Name	Data Sourc...	Barcode	Source	Collection ...	Location	Serov		ST	HC0 (indist...	HC2	HC5	HC10	HC20	HC50	HC100	HC2
SAL_DB8...			SLR1_8094	SRR12342991	SAL_DB8138...			Russia			238579	236467	236467	236467	236467	2	2	2	2

Uberstrain	✔	✎	Name	Data Sourc...	Barcode	Source	Collection ...	Location	Sero		ST	HC0 (indist...	HC2	HC5	HC10	HC20	HC50	HC100	H
SAL_DB8...			SLR1_8250	SRR12342993	SAL_DB8140...			Russia			238575	238575	238575	238575	238575	238575	87	87	12

Uberstrain	✔	✎	Name	Data Sourc...	Barcode	Source	Collection ...	Location	Serovar		ST	HC0 (indist...	HC2	HC5	HC10	HC20	HC50	HC10
SAL_IB81...			SLR1_8239	SRR15212458	SAL_IB8165...			Russia			278706	278706	278706	278706	278706	278706	87	87
SAL_IB81...			SLR1_8245	SRR15212459	SAL_IB8166...			Russia			278706	278706	278706	278706	278706	278706	87	87

Figure S4: Results of individual queries using different HC10 values (HC10 = 238586, HC10 = 236467, and HC10 = 238575).

As expected (see supplementary figure S2), for HC10 278706 we received two outbreak isolates SLR1_8245 and SLR1_8239.

We concluded that we did not reveal any close relationship of our isolates to each other or to other isolates (with the exception of SLR1_8245 and SLR1_8239) by core genome MLST in the EnteroBase as our isolates showed at least 10 allele differences by cgMLST.
